# Gamma-Klotho exhibits multiple roles in tumor growth of human bladder cancer

**DOI:** 10.18632/oncotarget.24628

**Published:** 2018-04-13

**Authors:** Shunta Hori, Makito Miyake, Yoshihiro Tatsumi, Yosuke Morizawa, Yasushi Nakai, Sayuri Onishi, Kenta Onishi, Kota Iida, Daisuke Gotoh, Nobumichi Tanaka, Kiyohide Fujimoto

**Affiliations:** ^1^ Department of Urology, Nara Medical University, Kashihara-Shi, Nara 634-8522, Japan

**Keywords:** urothelial carcinoma, gamma-Klotho, apoptosis, epithelial-mesenchymal transition, cancer prognosis

## Abstract

Alpha-Klotho (KLα) and beta-Klotho (KLβ) have recently been reported to correlate with cancer prognosis in some malignancies and we previously reported the association between KLα, KLβ, and urothelial carcinoma of the bladder (UCB), indicating that KLβ acts as a tumor promoter. However, the association between gamma-Klotho (KLγ) and cancer prognosis remains unclear. In the present study, we evaluated the association between KLγ and UCB. To evaluate the effect of KLγ on human bladder cancer cell lines *in vitro* assays were performed. Exogenous KLγ increased the ability of human bladder cancer cells to proliferate, migrate, invade, form colonies, and provide anchorage-independent growth potential. In *in vivo* assays, eighteen mice bearing xenografts inoculated using UM-UC-3, were randomly divided into three groups and treated with a small interfering RNA (siRNA) by intratumoral administration once a week for four weeks. Knockdown of KLγ with siRNA led to a dramatic change in tumor growth and suggested that KLγ had effects on tumor growth, including promotion of cell proliferation, inhibition of apoptosis, and enhancement of the epithelial-mesenchymal transition. To confirm the study, human tissue samples were used and patients were divided into two groups according to KLγ expression level. High expression of KLγ was significantly associated with higher stage and grade cancer and the presence of lymphovascular invasion compared to patients with lower expression of KLγ. Our results suggest that KLγ plays an important role in tumor invasion and progression and these results may lead to the development of new therapies and diagnostic methods for UCB.

## INTRODUCTION

Urothelial carcinoma of the bladder (UCB) is one of the most important health issues worldwide. In Japan, UCB is the eighth most common malignancy in men [1]. Approximately 70% of UCBs are diagnosed as non-muscle invasive bladder cancer (NMIBC) [2]. Although cancer-specific survival of patients with category Ta UCB is favorable, T1 NMIBC has the potential to progress to muscle invasive bladder cancer (MIBC), even with initial treatment including transurethral resection of bladder tumor (TURBT) and intravesical treatment. One of the biggest issues is that T1 UCB can be fatal, resulting from local progression or metastatic disease [3]. On the other hand, approximately 30% of UCBs are diagnosed as MIBC [4]. In spite of treatments, including radical cystectomy with or without neoadjuvant or adjuvant cisplatin-based chemotherapy, cancer-specific survival of patients with MIBC is not satisfactory [5]. With regard to advanced or metastatic UCB, mortality has remained unchanged for the last 30 years and the cancer-specific survival of patients with advanced or metastatic UCB is poor, despite treatment using cisplatin-based chemotherapy [6]. Therefore, the development of novel diagnostic and therapeutic targets is an important and urgent matter.

Gamma-Klotho (KLγ), also known as lactase-like protein, belongs to the glycosyl hydrolase 1 family and is a single-pass membrane protein containing 567 amino acids. KLγ also belongs to the Klotho subfamily [7]. The alpha-Klotho (KLa) gene was originally identified as an anti-aging gene and the beta-Klotho (KLb) gene encodes an amino acid sequence that is 41.2% identical to that of KLa [8, 9]. The Klotho family are co-factors of fibroblast growth factors (FGF) 19, FGF21, and FGF23, which regulate tissue-specific metabolic activity [10]. Recently, the association between cancer and KLa has been reported as a new biomarker for cancer [11]. Most of the reports have suggested that KLa plays the role of a tumor suppressor, including promotion of apoptosis and inhibition of transforming growth factor-b1 signaling, which is involved in the epithelial-to-mesenchymal transition (EMT) [12–16]. On the other hand, the role of KLb in cancer prognosis is still controversial [17–19]. Our previous report suggested that high expression of KLβ was an independent predictive factor for short progression-free survival of NMIBC and treatment with exogenous KLb increased proliferation, migration, transendothelial migration, and anchorage-independent growth *in vitro* [20]. With regard to KLγ, there are fewer reports about the association between KLγ and cancer. Previous reports showed that KLγ has an important role in the cell proliferation ability of colon cancer and the expression level of KLγ was associated with prognosis in triple negative breast cancer [21, 22]. In the present study, we focused on the clinical significance of KLγ, which could be a regulator of cancer progression for UCB.

## RESULTS

### KLγ promotes tumor growth and tumor invasiveness of urothelial cancer cells

**Figure 1:**
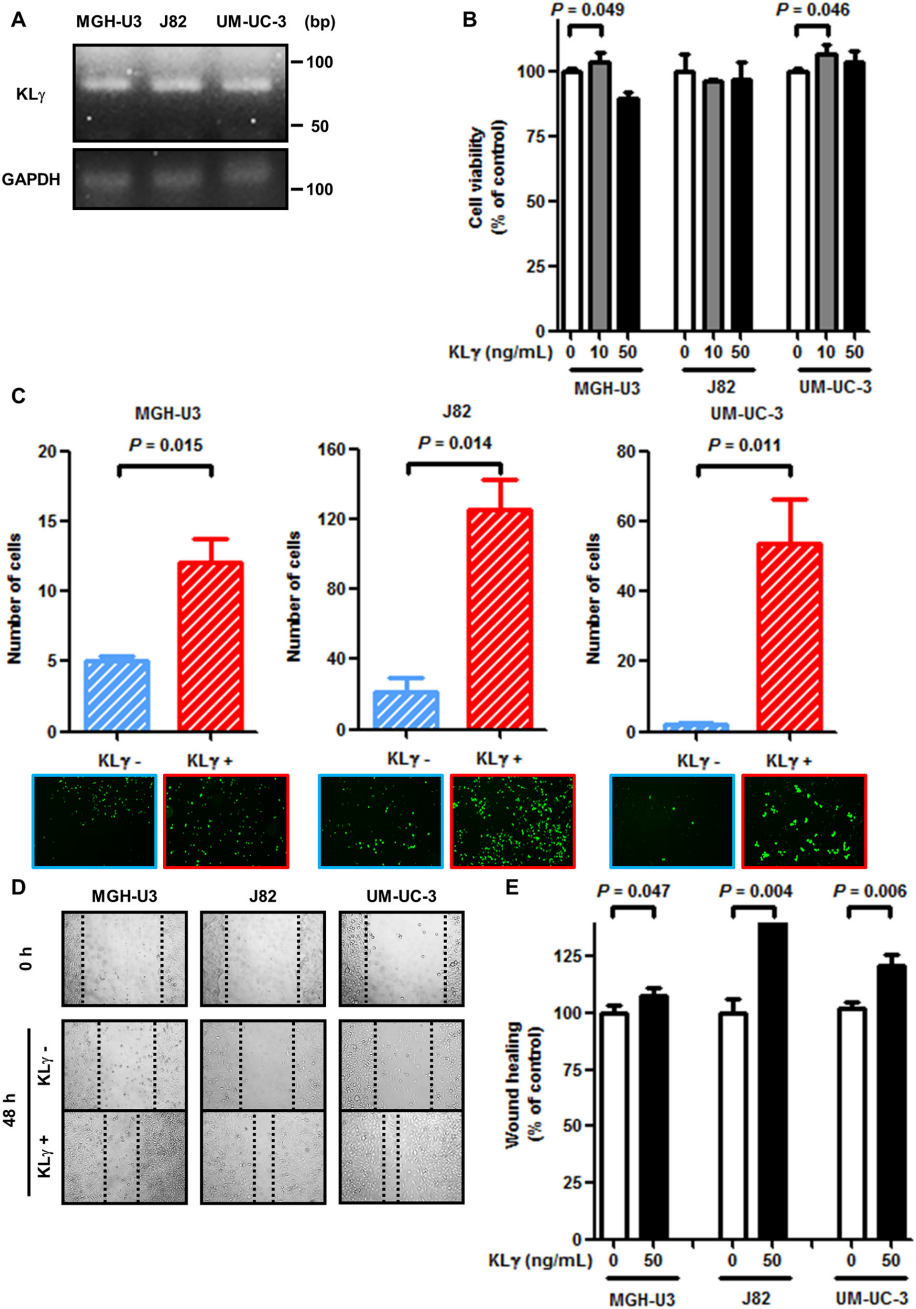
**(A)** MGH-U3, J82, and UM-UC-3 cells express KLγ RNA. **(B)** In the viability assay, treatment with 10 ng/mL exogenous KLγ increased the proliferation ability of MGH-U3 and UM-UC-3. **(C)** In the Matrigel invasion assay, treatment with exogenous KLγ increased the invasive ability of all three cell lines. **(D)** Representative images of wound healing assay for each cell line. After 48 h, migration ability was evaluated in each cell line. **(E)** In the wound healing assay, treatment with exogenous KLγ increased the migration ability of all three cell lines.

To check the expression level of KLγ in the three urothelial cancer cell lines, RT-PCR analysis was performed. All cell lines expressed endogenous KLγ mRNA (Figure 1A). Exogenous KLγ treatment at a concentration of 10 ng/mL promoted urothelial cancer cell proliferation by approximately 110% in MGH-U3 and UM-UC-3 cells (*P* = 0.049 and 0.046, respectively). In the remaining conditions, no effect was observed (Figure 1B). With regard to the Matrigel invasion assay, exogenous KLγ treatment enhanced invasiveness ability in MGH-U3, J82, and UM-UC-3 cells (*P* = 0.015, 0.014, and 0.011, respectively; Figure 1C).

Figure 1D shows representative images of cell migration with or without exogenous KLγ by the scratch wound healing assay. The result also revealed that exogenous KLγ treatment enhanced migration ability in MGH-U3, J82, and UM-UC-3 cells (*P* = 0.047, 0.004, and 0.006, respectively; Figure 1E). These results suggest that treatment with KLγ enhanced the cells’ ability to proliferate, invade, and migrate *in vitro*.

### KLγ treatment enhances colony formation potential and anchorage-independent growth

**Figure 2:**
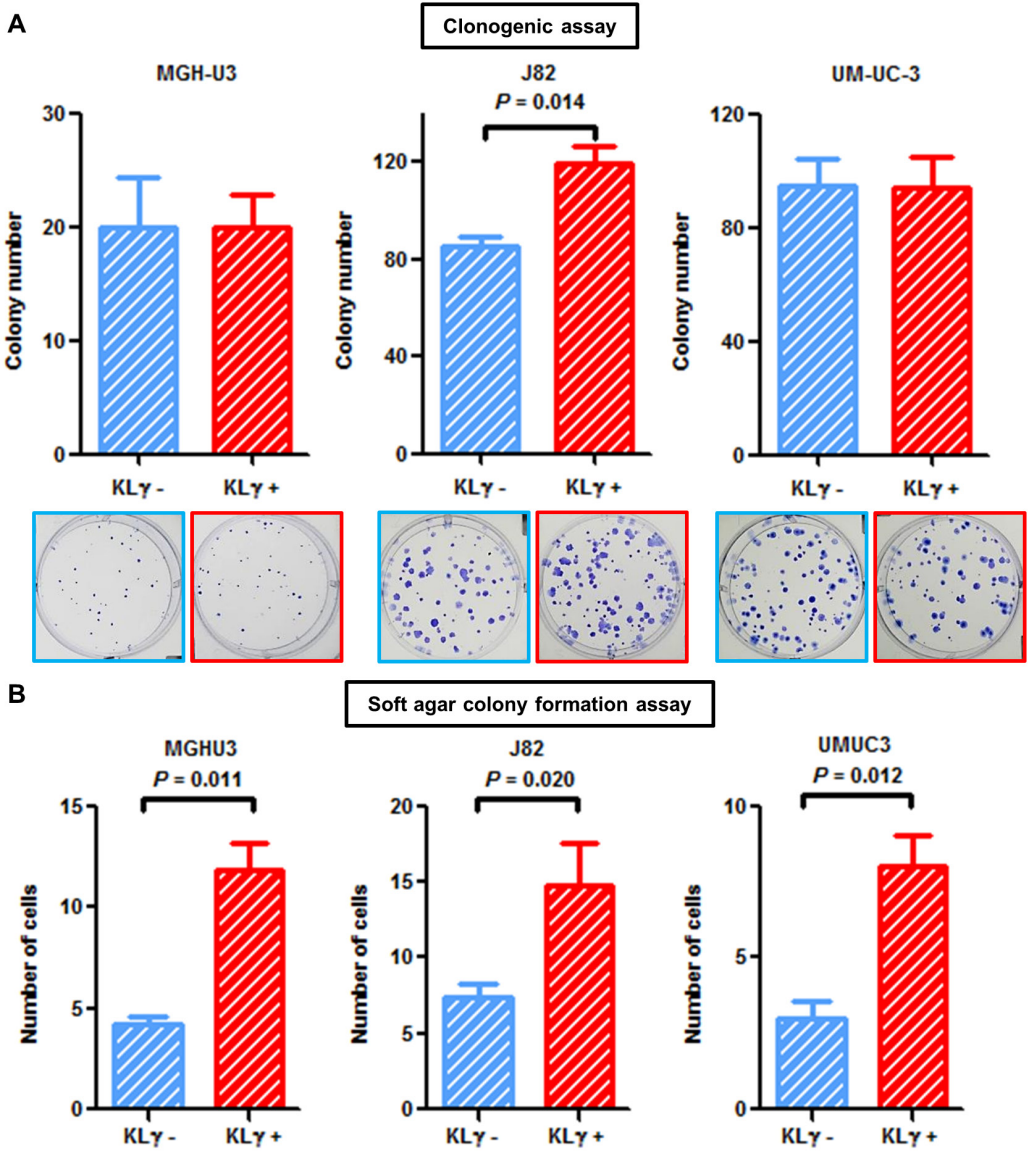
**(A)** In the clonogenic assay, treatment with exogenous KLγ increased colony formation ability in J82. **(B)** Soft agar assays with or without treatment with exogenous KLγ in three human urothelial carcinoma cell lines. The results showed that treatment with exogenous KLγ increased the anchorage-independent growth capability of all the three cell lines.

Colony formation ability was enhanced in J82 cells treated with exogenous KLγ (*P* = 0.014; Figure 2A). Cells were suspended in soft agar and incubated with or without 50 ng/mL KLγ. The number of colonies was counted one week after seeding. Evaluation on day 7 showed a notable increase in the colony formation ability of cells treated with KLγ in MGH-U3, J82, and UM-UC-3 cells (*P* = 0.012, 0.020, and 0.011, respectively; Figure 2B). The treatment with KLγ enhanced colony formation ability and anchorage-independent growth capability *in vitro*.

### KLγ siRNA treatment inhibits tumor growth *in vivo*

**Figure 3:**
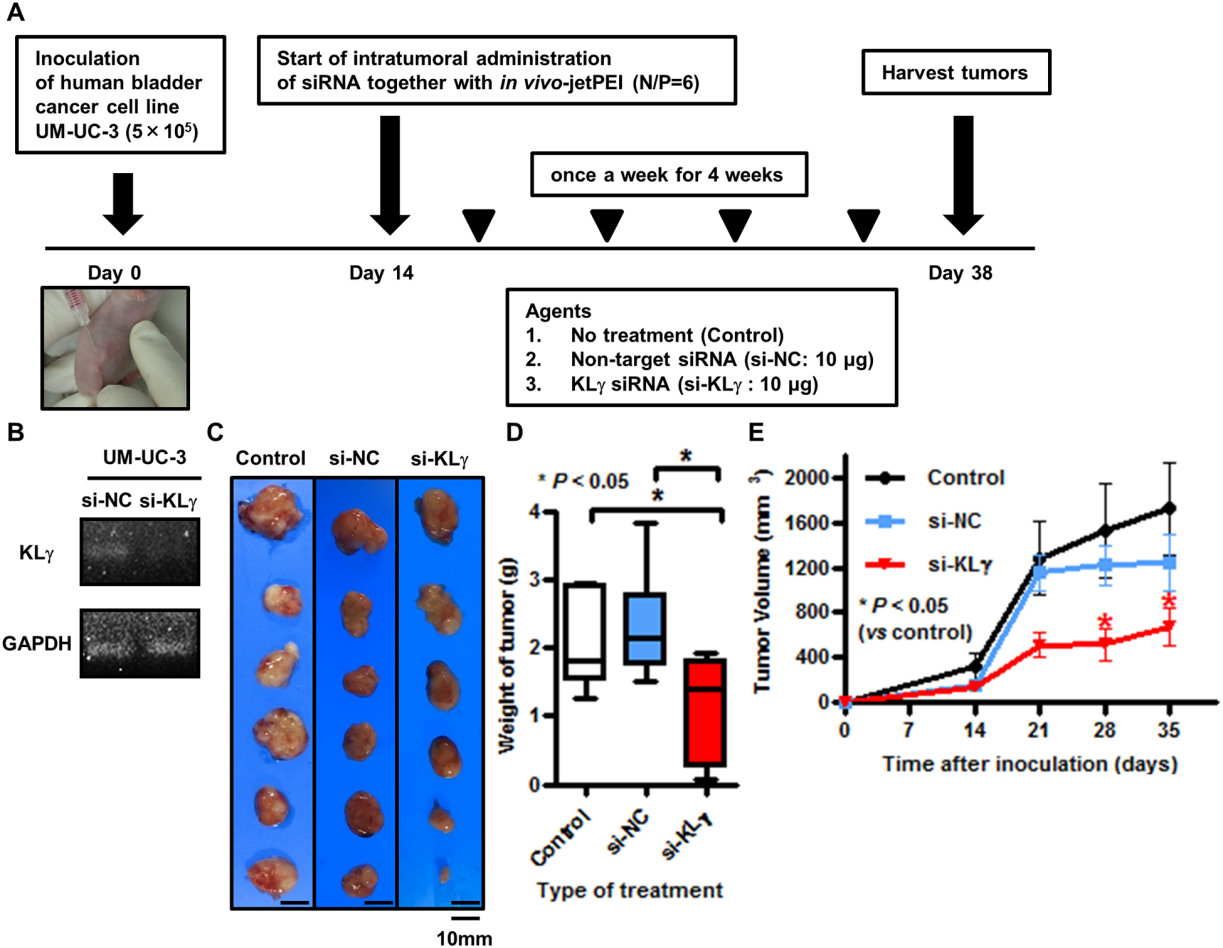
**(A)** Schematic diagram illustrating the study workflow. Mice were injected with UM-UC-3 cells (5×10^5^/tumor) together with Matrigel. Two weeks after inoculation, mice were randomly divided into three groups (n = 6 per group; control (no treatment), negative control siRNA, and human KLγ siRNA). Then, mice were treated once a week for 4 weeks. Three days after the last treatment, mice were euthanized and xenografts were harvested. **(B)** The treatment with KLγ siRNA suppressed the expression levels of KLγ mRNA in UM-UC-3, as measured by RT-PCR analysis. **(C)** The resected xenografts from treatment group were photographed. **(D)** Intratumoral treatment with KLγ siRNA caused significant resected xenograft weight loss compared to both the control group and the group of mice treated with negative control siRNA (Mann–Whitney *U* test; ^*^ = P< 0.05). **(E)** Tumor growth rate during treatment was significantly lower in mice treated with KLγ siRNA compared to both the control group and the group of mice treated with negative control siRNA (Mann–Whitney *U* test; ^*^ = P< 0.05).

Figure 3A shows a schematic representation of the *in vivo* study. In the *in vitro* study, we confirmed by RTPCR analysis that transfection with KLγ siRNA knocked down the expression level of KLγ mRNA in UM-UC-3 cells (Figure 3B). In the *in vivo* study, the treatments were well tolerated with no appreciable toxicity, including for body weight loss. Figure 3C shows representative images of xenografts in each group at the time of harvest. Xenografts of mice treated with KLγ siRNA were the smallest tumors among the three groups. Significant tumor weight loss was observed in mice treated with KLγ siRNA compared with the no treatment group (Figure 3D). The tumor growth rate during the treatment was significantly lower in mice treated with KLγ siRNA compared with the no treatment group (Figure 3E).

### KLγ siRNA treatment promotes EMT of bladder cancer cells

**Figure 4:**
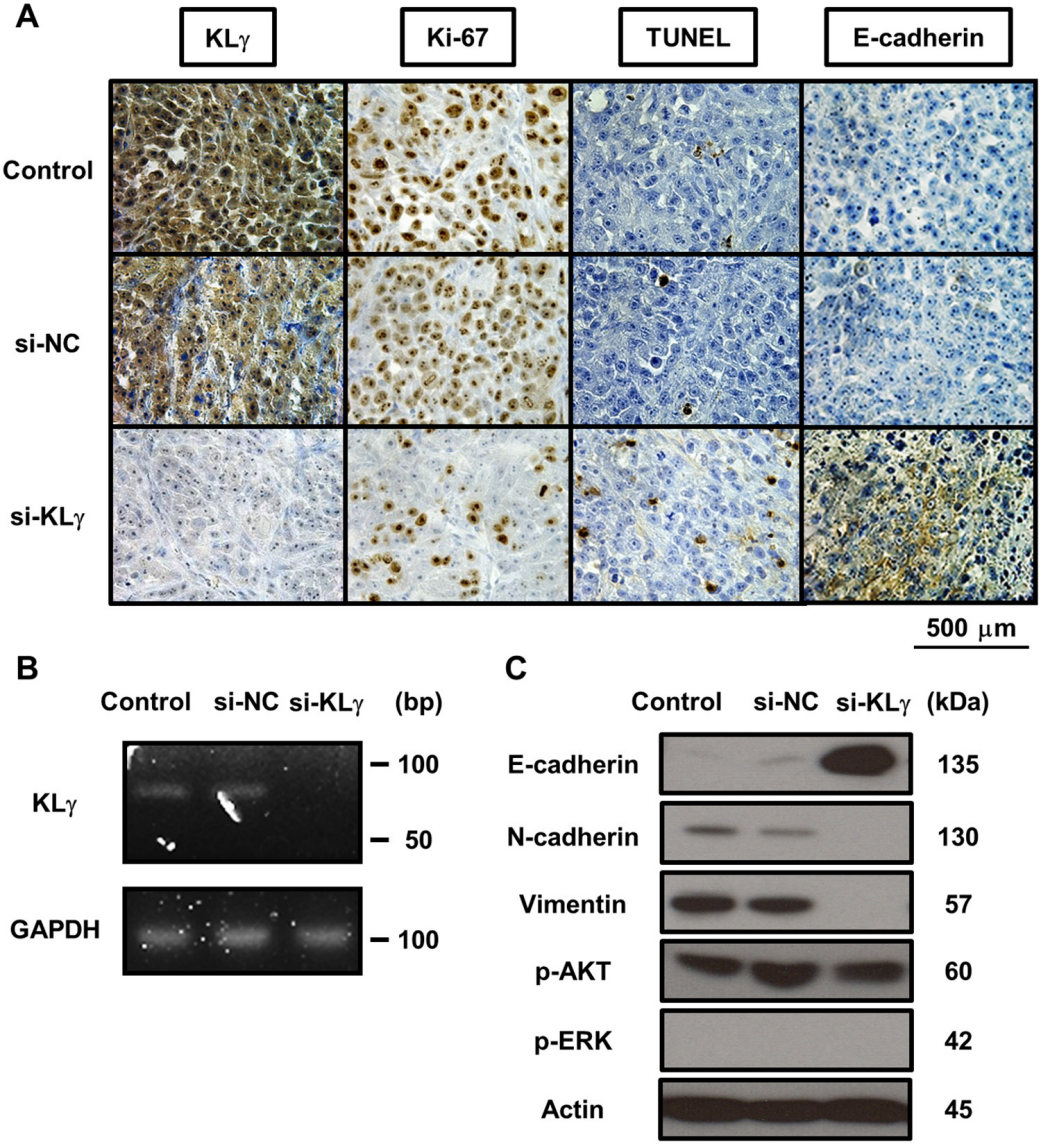
**(A)** Representative images of resected xenografts from each treatment group stained with four immunological markers. The expression levels of KLγ, Ki-67, TUNEL, and E-cadherin are noted. Expression levels of each marker in tumors of mice treated with KLγ siRNA compared to both the control group and the group of mice treated with negative control siRNA. The expression levels of KLγ and Ki-67 decreased in tumors of mice treated with KLγ siRNA. The expression levels of TUNEL and E-cadherin increased in tumors of mice treated with KLγ siRNA. **(B)** Treatment with KLγ siRNA suppressed the expression levels of KLγ mRNA in tumors of mice treated with KLγ siRNA, as measured by RT-PCR analysis. **(C)** Western blot analysis of protein extracted from resected xenografts in each treatment group. The expression levels of E-cadherin, N-cadherin, vimentin, phospho-AKT, phospho-ERK1/2, and actin (as a control) were noted. The expression level of E-cadherin was increased in tumors of mice treated with KLγ siRNA. The expression levels of N-cadherin and vimentin decreased in tumors of mice treated with KLγ siRNA.

Figure 4A shows representative images of xenograft tumors stained with each marker in each group. In the IHC analysis, xenografts of mice treated with KLγ siRNA showed significantly lower expression of KLγ compared with those of mice with no treatment (*P* = 0.0045; Supplementary Figure 1A). Xenografts of mice treated with KLγ siRNA showed significantly lower expression of Ki67 and an increase in apoptotic cells compared with those of mice with no treatment (*P* = 0.0050, Supplementary Figure 1B; *P* = 0.0063, Supplementary Figure 1C, respectively). Xenografts of mice treated with KLγ siRNA also showed significantly higher expression of E-cadherin compared with that of mice with no treatment (*P* = 0.0099; Supplementary Figure 1D). The higher expression level of KLγ was significantly correlated with higher expression of Ki67 and lower expression of TUNEL and E-cadherin (*P* = 0.0023, *P* = 0.025, and *P* = 0.0016, respectively; Table 1). To confirm the effect of treatment with KLγ siRNA, total RNA and cDNA was prepared from xenograft tumors and RT-PCR analysis was performed. KLγ mRNA level decreased in xenografts of mice treated with KLγ siRNA (Figure 4B). In addition, to investigate the involvement of EMT, the ERK1/2 pathway, and the Akt pathway, western blot analysis was performed. The concentration of E-cadherin increased in xenografts of mice treated with KLγ siRNA and the concentrations of N-cadherin and vimentin were decreased in this group. Phosphorylation levels of AKT and ERK1/2 were not affected significantly by the transfection of KLg siRNA (Figure 4C).

**Table 1:** Correlation between the expression level of KLγ and the expression levels of Ki-67/TUNEL/E-cadherin, as determined by IHC in the *in vivo* study

	Spearman r	*P* value
Ki67	0.79	0.0023
TUNEL	-0.64	0.025
E-cadherin	-0.81	0.0016

There was significant correlation between the expression level of KLγ and the others.

Abbreviations: KLγ, gamma-Klotho; TUNEL, terminal deoxynucleotidyl transferase-mediated dUTP nick end labeling; IHC, immunohistochemistry.

### The association of KLγ expression with clinicopathological variables in human bladder cancer

**Figure 5:**
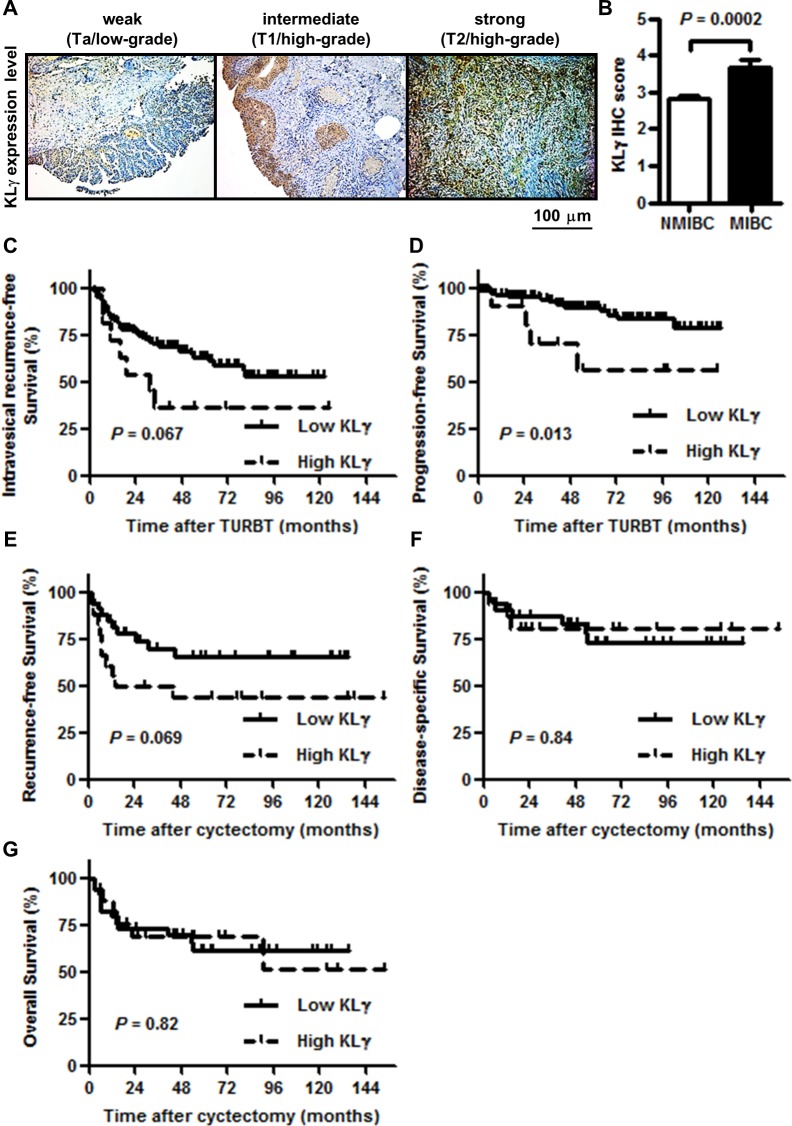
**(A)** KLγ was expressed in urothelial carcinomas of the bladder and the expression level gradually increased as tumors progressed to higher stages and grades. Scale bars, 100 μm. **(B)** Quantification of KLγ expression level in non-muscle invasive bladder cancer (NMIBC) versus muscle invasive bladder cancer (MIBC). The expression level of KLγ was significantly higher in MIBC than NMIBC. **(C)** Although patients with the high KLγ expression were tended to have the intravesical recurrence in non-muscle invasive bladder cancer, there was no significant difference. **(D)** Patients with the high KLγ expression significantly progressed to muscle invasive bladder cancer compared to patients with the low KLγ expression in non-muscle invasive bladder cancer. **(E)** Although patients with the high KLγ expression were tended to have the recurrence in muscle invasive bladder, there was no significant difference. **(F)** There was no significant difference of disease-specific survival between patients with low KLγ expression and those with high KLγ expression in muscle invasive bladder cancer. **(G)** There was no significant difference of overall survival between patients with low KLγ expression and those with high KLγ expression in muscle invasive bladder cancer.

To investigate the association between the expression levels of KLγ in bladder tumor tissues and clinicopathological variables, IHC analysis for KLγ expression was performed. Figure 5A shows representative images of weak, intermediate, and strong expression of KLγ. KLγ expression was higher in MIBC than that in NMIBC (*P* = 0.0002; Figure 5B). Table 2 shows the clinicopathological backgrounds of a cohort of 151 NMIBC and 54 MIBC patients and comparisons of the variables with high and low KLγ expression. The pathological data, such as tumor category, tumor grade, and lymphovascular invasion (LVI), were significantly different between patients with low and high KLγ expression. The preoperative status including performance status and comorbidity were not correlated with the KLγ expression. Thirty-one patients out of 205 died during the follow-up period and there were no association between cause of death and KLγ expression.

**Table 2:** Patients’ clinicopathological background

Variables		Number of patients	KLγ expression		*P* value
			Low	High	
Total		205	176	29	
Gender					0.78 ^†^
	Male	173	149	24	
	Female	32	27	5	
Age (at initial TURBT)					0.26 ^‡^
	Median (IQR)	71 (34 - 94)	71 (34 - 93)	72 (52 - 83)	
ECOG-PS (at initial TURBT)					0.23 ^†^
	0	190	165	25	
	1	9	6	3	
	2	6	5	1	
Charlson comorbidity index					0.83 ^†^
	0	138	119	19	
	≥ 1	67	57	10	
Follow up (months)					0.62 ^‡^
	Median (IQR)	52 (3 - 154)	52 (3 - 135)	53 (3 - 154)	
T category					0.0003 ^†^
	Ta	65	61	4	
	T1	72	67	5	
	Tis	14	12	2	
	T2	29	20	9	
	T3	18	12	6	
	T4	7	4	3	
Tumor Grade (2004 edition)					0.0049 ^†^
	High	137	111	26	
	Low	68	65	3	
Tumor Grade (1973 edition)					0.0010 ^†^
	G1	21	19	2	
	G2	86	82	4	
	G3	98	75	23	
INF					0.46 ^†^
	a/b	165	143	22	
	c	40	33	7	
Concomitant CIS with pTa/1/2/3/4 (n=191)					0.51 ^†^
	Yes	63	56	7	
	No	128	108	20	
LVI with pT1/2/3/4 (n=126)					0.039 ^†^
	Yes	62	46	16	
	No	64	57	7	
Cause of death					0.41 ^†^
	Bladder cancer	21	16	5	
	Another cancer	2	1	1	
	Pneumonia	3	3	0	
	Peritonitis	1	1	0	
	Chronic renal failure	1	0	1	
	Unknown	3	2	1	

Patients with high KLγ expression showed high stage, high grade cancer and the presence of lymphovascular invasion.

Abbreviations: KLγ, gamma-Klotho; TURBT, transurethral resection of bladder tumor; IQR, interquartile range; ECOG-PS, Eastern Cooperative Oncology Group performance status; INF, infiltration; CIS, carcinoma *in situ*; LVI, lymphovascular invasion; ^†^ Chi-square test or Fisher's exact test; ^‡^ Mann–Whitney *U* test.

### The expression level of KLγ is associated with progression-free survival of patients with NMIBC

Of the 205 patients, 21 (10.2%) died of UCB at a median of 50 months after initial TURBT. Of those 21 patients, 8 patients were diagnosed as NMIBC primarily and progressed to MIBC. The remaining 13 patients were diagnosed as MIBC primarily, underwent radical cystectomy, and then had a recurrence in lymph node, lung, or bone. Of the 151 NMIBC patients, 53 (35.1%) had an intravesical recurrence at median 35.5 months after initial TURBT and 18 (11.9%) progressed to MIBC at median 52.0 months after initial TURBT. With regard to the intravesical recurrence- and progression-free survival in patients with high or low KLγ expression, intravesical recurrence was tended to be more in patients with high KLγ expression and intravesical progression significantly increased in patients with high KLγ expression (*P* = 0.067, Figure 5C; *P* = 0.013, Figure 5D, respectively). In contrast, of the 54 MIBC patients, 20 (37.0%) had a recurrence at median 26.0 months after radical cyctectomy, 10 (18.5%) died of UCB at median 46.5 months after radical cyctectomy, and 8 (14.8%) died of other causes, such as lung cancer, pneumonia, and chronic kidney disease, at median 54.0 months after radical cyctectomy. With regard to the recurrence-free, disease-specific, and overall survival in patients with high or low KLγ expression, there was no significant difference between the two groups (*P* = 0.069, Figure 5E; *P* = 0.84, Figure 5F; *P* = 0.82, Figure 5G, respectively).

Table 3 shows the univariate and multivariate analysis of prognostic factors for the intravesical progression-free survival in NMIBC patients. The univariate analysis revealed that tumor grade, infiltration (INF) pattern, the presence of carcinoma *in situ*, the presence of LVI, and high KLγ expression was the predictive factors for the intravesical progression (hazards ratio [HR] = 3.0, 95% confidence interval [CI] 1.0-9.1; *P* = 0.05; HR = 6.6, 95% CI 2.5-17.4, *P* < 0.0001; HR = 2.8, 95% CI 1.1-7.5, *P* = 0.034; HR = 3.2, 95% CI 1.3-7.9, *P* = 0.014; HR = 3.6, 95% CI 1.2-10.9, *P* = 0.013, respectively). The multivariate analysis showed that INF and high KLγ expression were independent prognostic factors for the intravesical progression-free survival in NMIBC patients. (HR = 7.8, 95% CI 1.3-44.9; *P* = 0.022; HR = 4.3, 95% CI 1.2-10.9; *P* = 0.025).

**Table 3:** Cox regression analysis of prognostic factors for overall survival

Variables		Univariate analysis			Multivariate analysis		
		HR	95% CI	*P* value	HR	95% CI	*P* value
Gender				0.39			
	Male	1					
	Female	1.7	0.5 - 6.0				
Age				0.27			
	< 70	1					
	≥ 70	1.7	0.7 - 4.6				
T category				0.099			
	Ta	1					
	T1	2.5	0.8 - 7.8				
	Tis	4.5	1.1 - 18.1				
Tumor Grade				0.05			0.61
	Low	1			1		
	High	3	1.0 - 9.1		0.58	0.07 - 4.8	
INF				< 0.0001			0.022
	No	1			1		
	Yes	6.6	2.5 - 17.4		7.8	1.3 - 44.9	
CIS				0.034			0.45
	No	1			1		
	Yes	2.8	1.1 - 7.5		1.8	0.4 - 8.3	
LVI				0.014			0.59
	No	1			1		
	Yes	3.2	1.3 - 7.9		1.7	0.3 - 10.4	
KLγ expression				0.013			0.025
	Low	1			1		
	High	3.6	1.2 - 10.9		4.3	1.2 - 15.3	

The high expression level of KLγ was not an independent poor prognostic factor.

Abbreviations: KLγ, γ–klotho; INF, infiltration; CIS, carcinoma *in situ*; LVI, lymphovascular invasion; HR, hazard ratio; CI, confidence interval.

## DISCUSSION

Our previous report suggests that KLb acts as a tumor promoter in human UCB and that KLa does not have a significant role as a tumor suppressor or promoter in human UCB [20]. We hypothesized that KLb plays a role as a tumor promoter, together with FGF21 or FGF19. Although additional experiments were performed, we could not demonstrate an association between KLb and the FGF receptor (FGFR) pathways involving FGF21 and FGF19. Therefore, we focused on another co-factor of FGFs, named KLγ, which predominantly acts with FGF19 and FGFR4 [23]. FGF/FGFR signaling plays an important role in tumor progression, including tumor proliferation, cell differentiation, and angiogenesis [24–26]. Thus, we hypothesized that KLγ had a role as a tumor promoter in UCB through the activation of FGF/FGFR signaling.

The present study revealed that in an *in vitro* study, treatment with exogenous KLγ increased the ability of invasion, migration, colony formation, and anchorage-independent growth. With regard to cell viability assay, dose dependent manner was not observed. Although the reason is not clear, the solvent of recombinant-KLγ protein might contain harmful materials. In the *in vivo* study, tumor growth was significantly suppressed by treatment with KLγ siRNA, suggesting that KLγ could act as a tumor promoter. The present study also showed that patients with high KLγ expression had a significantly higher stage and grade of UCB and a higher risk rate of the intravesical progression. In multivariate analysis, high KLγ expression was an independent prognostic factor for progression-free survival in patients with NMIBC. Although the result of *in vitro* study suggested that high expression level of KLγ affected INF, there was no significant difference between patients with high expression level of KLγ and those with low expression level of KLγ. This might be because the number of patients was apparently small.

At present, most of our understanding regarding the functional roles of FGFRs and their signaling pathways has been derived mainly from the study of FGFR1, FGFR2, and FGFR3. There are only limited reports about FGFR4. Recently, there have been several reports about the association between FGF19 and FGFR4 signaling and cancer prognosis. In hepatocellular carcinoma (HCC), the expression level of FGF19 in HCC tissues was significantly higher compared with noncancerous liver tissues and tumor FGF19 mRNA expression was an independent prognostic factor for mortality. In addition, FGF19 and FGFR4 signaling had an effect on HCC resistance to sorafenib therapy through the inhibition of reactive oxygen species generation and apoptosis [27, 28]. Tiong *et al.* suggested that FGF19 and FGFR4 signaling mediated the viability of cancer cells through activation of the PI3K/AKT pathway in breast cancer [29]. Hu *et al.* also suggested that patients with high expression of both FGF19 and FGFR4 had significantly poor prognoses for ovarian cancer and that FGF19 and FGFR4 signaling could promote ovarian cancer proliferation and invasion through the AKT-MAPK signaling pathway [30]. In prostate cancer, FGF19/FGFR signaling promotes cancer progression along with the presence of KLa and/or KLb as co-factors [18]. There have been no reports about the oncological function of FGF19/FGFR4 signaling in UCB.

In the *in vivo* study, treatment with KLγ siRNA suppressed tumor growth rate, indicating that KLγ has some role in promoting tumor cell growth, including enhancement of the cell cycle and inhibition of apoptosis. Although we expected that this phenomenon was involved in the activation of the ERK1/2 signaling pathway or the AKT signaling pathway, we could not determine a direct relationship between KLγ and those signaling pathways. Previous reports suggested that FGF/FGFR signaling affects the ability of proliferation and apoptosis in tumor cells via the activation of the ERK1/2 or AKT signaling pathways [31, 32]. Kim *et al*. reported that KLγ is an important factor for cell proliferation and that the presence of various KL members affects the degree of activation of signaling pathways in colon cancer [21]. Trošt *et al*. showed that, in patients with triple negative breast cancer, the expression level of KLγ was high and correlates with poor progression, that KLγ was a necessary factor for cell survival, and that depletion of KLγ resulted in cell cycle arrest, apoptosis, and persistent activation of the ERK1/2 signaling pathway [22]. Therefore, we hypothesize that KLγ plays a role in the promotion of bladder cancer and that the balance of existing KLs affects downstream signaling, including that of FGF and FGFR.

With regard to the epithelial-to-mesenchymal transition (EMT), there have been several reports indicating the association of KLs and FGF/FGFR signaling. KLa has a role in inhibiting TGF-β1-induced EMT and FGF19/FGFR4 signaling induces EMT in cholangiocarcinoma. In HCC, FGF19/FGFR4 signaling enhances EMT via the GSK3β/β-catenin signaling pathway [14, 33, 34]. Also with respect to UCB, EMT is involved in tumor progression and metastasis and expression levels of FGFRs are correlated with expression levels of EMT marker such as E-cadherin and vimentin [35–37]. Thus, we hypothesized that high expression of KLγ promoted EMT in bladder cancer, resulting in poor prognosis. In the *in vivo* study, treatment with KLγ siRNA suppressed a decrease in the expression of E-cadherin and enhanced the expression of N-cadherin and vimentin. This result suggests that the depletion of KLγ in bladder cancer leads to cadherin switching, resulting in the suppression of EMT. Although the mechanism for EMT induction is not clear, KLγ might be correlated with the induction of EMT in bladder cancer. We speculate that the EMT related with KLγ is induced by FGF19/FGFR4 signaling because KLγ is a co-factor of FGF19.

**Figure 6:**
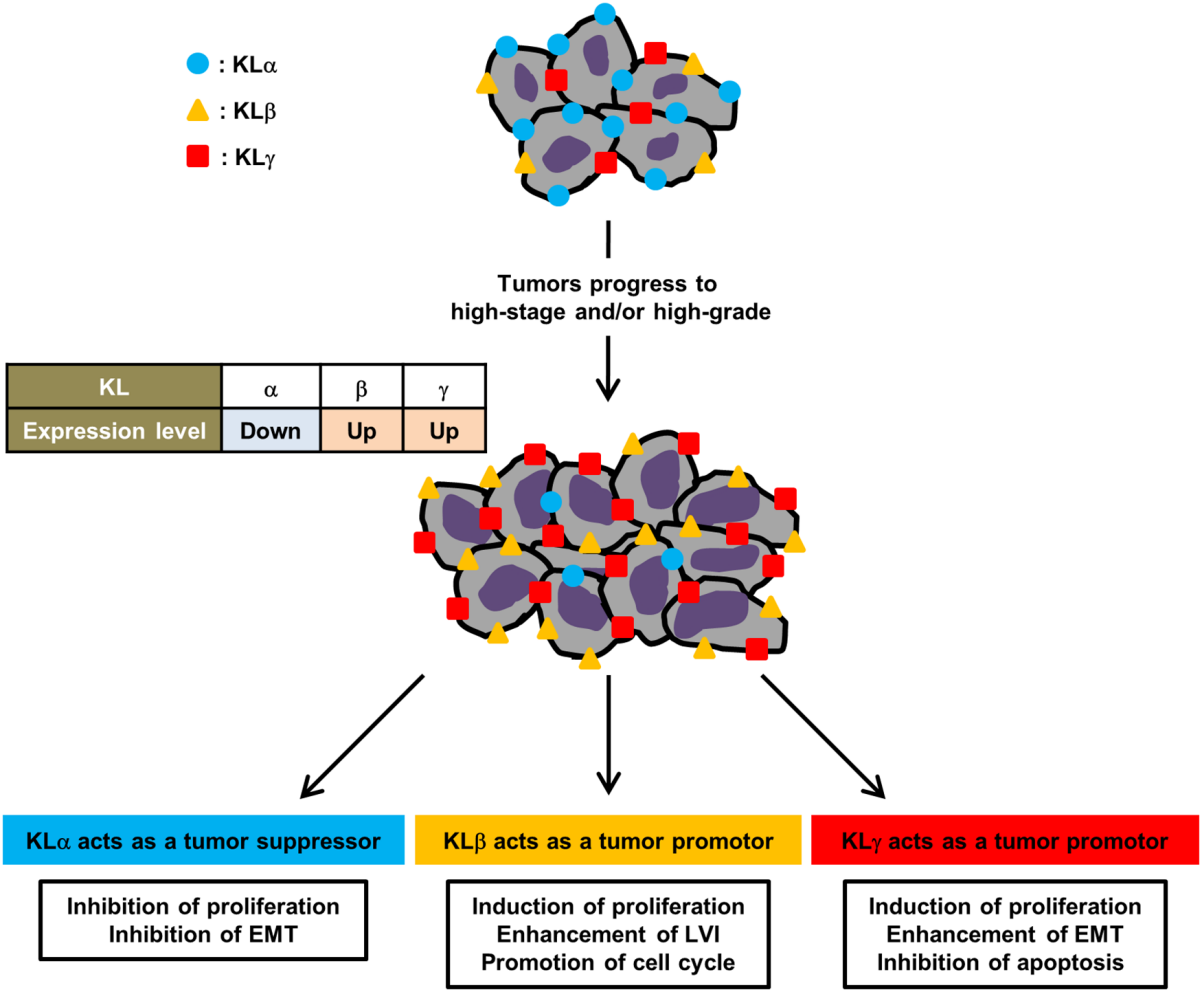
Roles of klotho family in bladder cancer In urothelial carcinoma of the bladder, along with increase of tumor grade the expression level of KLα decrease and the expression levels of KLβ and KLγ increase. KLs are orchestrating to affect cancer progression. KLa acts as a tumor suppressor mediated by inhibition of the IGF-1/insulin signaling pathway and EMT. KLb acts as a tumor promotor mediated by regulation of FGF4 signaling pathway, phosphorylation of ERK1/2 and FRS2. Similarly, KLγ acts as a tumor promotor mediated by regulation of FGF4 signaling pathway, ERK1/2 pathway, and EMT.

To our knowledge, the present study is the first report regarding the association between KLγ and bladder cancer prognosis. The expression of KLγ was higher in patients with higher stage, higher grade cancer, and presence of LVI compared to those patients with lower stage, lower grade cancer, and an absence of LVI. The expression level of KLγ was an independent prognostic factor for the intaravesical progression in patients with NMIBC. In addition, *in vitro* study the treatment with exogenous KLγ showed increase in tumor progression capability such as invasion, migration, and colony formation and *in vivo* study the treatment with KLγ siRNA showed suppression of tumor growth. Therefore we believe that KLγ acts as a promoter of UCB. Together with our previous study of UCB, KLa, KLb, and KLγ may be correlated with tumor growth. Our results suggested that the KLa expression level decreased along with increase of tumor grade, contrary the KLb and KLγ expression levels increased along with increase of tumor grade. KLa has tumor-suppressing roles on cell proliferation and survival, resulting from inhibition of the IGF-1/insulin signaling pathway and EMT. KLb and KLγ act as a tumor promotor. KLb affects FGF4 signaling pathway, phosphorylation of ERK1/2 and FRS2, resulting in enhancement of cell proliferation or inhibition of cancer cell apoptosis. KLγ also might affect FGF4 signaling pathway, ERK1/2 pathway and EMT, resulting in cancer prognosis. In addition, the KLs expression levels are correlated with each other and this status might affect cancer prognosis (12, 15, 17, 20-22). Figure 6 shows the association on roles of each KL in UCB.

This study has some limitations. Firstly, the direct relationship between KLγ as a co-receptor of FGFR and FGF/FGFR signaling could not be elucidated in this study. We attempted to show the association between KLb and FGF/FGFR signaling in a previous study; however, human bladder cancer samples were not stained with anti-FGF and anti-FGFR antibodies. Further, experiments using natural carcinogenesis and a KLγ knockdown mouse model of bladder cancer are needed to elucidate the involvement of KLγ in bladder cancer. Secondly, cytokines involved in cell proliferation, apoptosis, and EMT were not evaluated in this study. Further, experiments are needed to reveal the association between KLγ and these mechanisms of tumorigenesis. Thirdly, there might be variations about tumor injection, tumor growth, and intratumoral administration. These variations might affect the results of *in vivo* study. Fourthly, although KLγ was an independent prognostic factor for the intaravesical progression in patients with NMIBC, KLγ was not a poor prognostic factor for patients with MIBC. We need to increase the number of patients to reveal the association between KLγ and UCB. Careful interpretation needs to be done in a confirmation study using clinicopathological data.

In conclusion, our findings suggested that KLγ plays a pivotal role in the growth of human UCB. The expression level of KLγ was associated with cell proliferation, apoptosis, and EMT, creating a milieu favorable to the survival and expansion of tumors. Improved knowledge about the actual involvement of KLγ, together with KLa and KLb, is expected to facilitate the search for new therapies and diagnostic methods for UCB.

## MATERIALS AND METHODS

### Cell lines and reagents

The human urothelial carcinoma cell lines MGH-U3, J82, and UM-UC-3 were used in this study. MGH-U3 was a gift from Dr. H. LaRue (Laval University Cancer Research Centre, Quebec, Canada). J82 and UMUC-3 were purchased from the American Type Culture Collection (ATCC, Manassas, VA, USA). The cell lines were maintained in RPMI-1640 medium (Nacalai Tesque, Kyoto, Japan) supplemented with 10% fetal bovine serum (FBS; JRH, Tokyo, Japan) and 1% penicillin and streptomycin (Thermo Scientific, Yokohama, Japan) in a standard humidified incubator at 37°C in an atmosphere of 5% CO_2_. Recombinant human KLγ protein was purchased from Abnova (H00197021-P01; Taipei, Taiwan) and diluted in sterile saline solution according to the manufacturer's instructions. Sterile phosphate-buffered saline (PBS) was used as a control.

### Quantitative reverse transcription-polymerase chain reaction (RT-PCR)

Quantitative RT-PCR was performed to measure the expression levels of KLγ RNA in each cell line and resected tumors. For *in vitro* study, cells were seeded in 6-well plates at a density of 1×10^5^ cells/well in growth medium and incubated for 24 h. RNA was extracted using an RNeasy Mini kit (Qiagen, Valencia, CA, USA) according to the manufacturer's instructions. For the *in vivo* study, after homogenization of tumors, RNA was extracted using a QIAamp RNA Blood Mini kit (Qiagen, Valencia, CA, USA) according to the manufacturer's instructions. Conversion to cDNA was achieved using a High Capacity cDNA Reverse Transcription kit (Life Technologies, Carlsbad, CA, USA). Quantitative RT–PCR was carried out using the cDNA, 0.2 μM of each primer, and 10 μL of AmpliTaq Gold® PCR Master Mix (Applied Biosystems, Foster City, CA, USA) under the following conditions: denaturation at 95°C for 10 min; 40 cycles of denaturation at 95°C for 15 sec; annealing and a final extension at 60°C for 1 min. PCR products were then electrophoresed in a 1.5% agarose gel and visualized by a transilluminator. Subsequent to verifying the mRNA expression of KLγ, semi-quantitative RT-PCR for this gene was performed in each cell line and resected tumors. Glyceraldehyde 3-phosphate dehydrogenase (GAPDH) was used as a control.

### Cell viability assays

Cell proliferation assays were performed to examine the effects of the exogenous recombinant human KLγ. Cells were seeded in a 96-well plate at a density of 2,000 cells/well in serum-free medium, incubated for 24 h, and treated with three different conditions of exogenous KLγ (0, 10, or 50 ng/mL) for 48 h at 37°C in an atmosphere of 5% CO_2_. Cell Counting Kit-8 (Dojindo, Kumamoto, Japan) was used to measure the number of viable cells according to the manufacturer's instructions.

### Matrigel invasion assay

To evaluate whether cell migration ability would increase with exogenous KLγ, the Matrigel invasion assay was performed using a FluoroBlok insert system. The insert membrane chambers were coated with 100 μL of Matrigel (Corning Incorporated, Corning, NY, USA) and incubated at 37°C in an atmosphere of 5% CO_2_. Cells were seeded in the upper chambers at a density of 2.5×10^4^ cells/well in serum-free medium and incubated at 37°C in an atmosphere of 5% CO_2_ with or without 50 ng/mL KLγ. After 48 h of incubation, non-invading cells in the upper chambers were removed, the invading cells in the lower chambers were stained with Calcein AM (Promokine, Heidelberg, Germany), and the cells were immediately examined under a fluorescence microscope (Leica DMI 4000B, Wetzlar, Germany).

### Wound healing assay

To evaluate the effect of exogenous KLγ on cell migration ability, the scratch wound healing assay was performed. Cells were seeded in a 12-well plate at a density of 5×10^4^ cells/well and grown to approximately 90% confluency at 37°C in an atmosphere of 5% CO_2_. Each plate was scratched with 10-100-μL pipette tips, rinsed twice with PBS, and then incubated in serum-containing medium with or without 50 ng/mL KLγ. At 0 and 48 h incubation, the width of the wound was measured at four randomly selected points and the width of the wound at 0 h was compared with the width at 48 h.

### Clonogenic assay

To evaluate whether exogenous KLγ increases colony formation, a colony formation assay was performed. Cells were seeded in a 6-well plate at a density of 2,000 cells/well in serum-containing medium and incubated at 37°C in an atmosphere of 5% CO_2_ with or without 50 ng/mL KLγ. After two weeks, colonies were fixed with methanol and stained with 0.1% crystal violet (Sigma-Aldrich, St Louis, MO, USA) according to the manufacturer's instructions. The number of visible colonies was measured for each well.

### Soft agar colony formation assay

To examine anchorage-independent growth, a soft agar colony formation assay was performed. A base agar layer was prepared using a 1.2% agar solution (Difco, Franklin Lakes, NJ, USA) mixed with an equal volume of 2× Dulbecco's modified Eagle's medium (DMEM; Sigma-Aldrich) with 20% FBS in a 24-well culture plate. Cell suspensions (2.5×10^4^ cells/mL) were prepared and mixed with both the 1.2% agar solution and 2× DMEM with 20% FBS in the same manner as described above. Exogenous KLγ (50 ng/mL) or PBS as a control was added to each well and incubation was carried out at 37°C in an atmosphere of 5% CO_2_. A week after seeding, the number of growing colonies was counted under a microscope.

### Animals

Animal care was in compliance with the recommendations of The Guide for Care and Use of Laboratory Animals (National Research Council) and this study was approved by the animal facility committee at Nara Medical University (protocol ID: 11896). Female athymic BALB/c nu/nu mice, 6 to 8 weeks old, were purchased from Oriental Bio Service (Kyoto, Japan). All mice were maintained under pathogen-free conditions and provided with sterile food and water.

### Transfection of small interfering RNA (siRNA)

For the *in vitro* study, UM-UC-3 cells were transfected with synthesized KLγ siRNA or negative control siRNA (Invitrogen, Life Technologies) with 50 pmol of siRNA and 5 μL of Lipofectamine 2000 (Life Technologies) using 6-well plates according to the manufacturer's instructions. After 48 h of transfection, RNA was extracted and expression of KLγ was measured in each cell line.

### Xenograft model and intratumoral treatment

After allowing mice to acclimate to our facility for one week, bladder cancer cells (UM-UC-3; 5×10^5^/tumor) in 50 μL RPMI-1640 medium, together with 50 μL of Matrigel (Corning Incorporated), were injected into flank of each mouse. Two weeks after cell inoculation, when the tumors reached 5 mm in diameter, we randomly divided the mice into three groups (n = 6 mice per group): control (no treatment), negative control siRNA (10 μg of non-target siRNA mixed with 1.2 μL of *in vivo*-jetPEI reagent with an N/P ratio of 6, according to the manufacturer's protocol), and human KLγ siRNA (10 μg of siRNA with 1.2 μl of *in vivo*-jetPEI reagent with an N/P ratio of 6, according to the manufacturer's protocol). Treatment was then initiated. KLγ siRNA therapy was administered intratumorally once a week for four weeks. To ensure optimal delivery to xenograft tumors, *in vivo*-jetPEI reagent (Polyplus-transfection Inc., New York City, NY, USA) was used in conjunction with siRNA [38]. Tumor diameters were measured once a week with electronic calipers and tumor volumes were calculated using the following formula: {(width)^2^ × length}/2 (mm^3^). Three days after the last treatment, all mice were euthanized by exsanguination under anesthesia with isoflurane and tumors were harvested for the following experiments.

### Immunohistochemistry (IHC) analysis of xenograft tumors

Tumors were examined by IHC staining analysis as previously described [20]. Briefly, anti-KLγ antibody, antiKi-67 antibody (clone MIB-1, ready to use; Dako, Japan), and anti-E-cadherin antibody (3195; rabbit monoclonal, dilution 1/1000; Cell Signaling Technology, Beverly, MA, USA) were used for IHC analysis. Moreover, to identify apoptotic cells by terminal deoxynucleotidyl transferase-mediated dUTP nick end labeling (TUNEL) assay, apoptotic cells in the xenografts were detected using a TUNEL apoptosis detection kit (R&D Systems, Minneapolis, MN, USA) according to the manufacturer's instructions.

### Western blot analysis of xenograft tumors

Protein was extracted from resected tumors and western blot analysis was performed as previously described [39]. Briefly, for protein extraction, tumor samples were minced and incubated in lysis buffer (250 mmol/L Tris-HCl (pH 6.8), 2% SDS, and 10% glycerol) and protein inhibitor cocktail (Sigma-Aldrich). The membranes were incubated for 1 h with primary anti-Ecadherin rabbit monoclonal antibody (dilution 1/1000), anti-N-cadherin mouse monoclonal antibody (dilution 1/500), anti-vimentin rabbit monoclonal antibody (dilution 1/500), anti-phospho-AKT rabbit monoclonal antibody (dilution 1/1000), anti-phospho-ERK1/2 rabbit monoclonal antibody (dilution 1/1000), or anti-actin mouse monoclonal antibody (dilution 1/10,000) as an internal loading control, followed by 1 h with horseradish peroxidase-conjugated anti-rabbit IgG (1:5,000) or anti-mouse IgG antibody (1:20,000) (Santa Cruz Biotechnology).

### IHC of human samples

To investigate the expression levels of KLγ, IHC was performed as previously described [20]. Paraffin-embedded tissues obtained from all 205 patients at the initial TURBT were used in this study to examine the association between KLγ expression level and clinicopathological variables. Briefly, the slides were incubated overnight at 4°C with anti-KLγ antibody (sc-137559; goat polyclonal, dilution 1/500; Santa Cruz Biotechnology, Santa Cruz, CA, USA). The slides were counterstained with hematoxylin, dehydrated, and mounted on a cover slide. We evaluated each slide using IHC scores (IHC score = intensity score + population score; intensity: none = 0, low = 1, intermediate = 2, and high = 3; population: none = 0, 0–25% = 1, 25–50% = 2, 50–75% = 3, and 75–100% = 4). The KLγ expression was categorized as low or high according to the IHC score as follows: low = IHC score ≤ 4; high = IHC score 5 or 6. Then the intravesical recurrence-free survival and progression-free survival in NMIBC patients were evaluated. We defined the intravesical progression as to progress from NMIBC to MIBC. Recurrence-free survival, disease-specific survival, and overall survival in MIBC patients were also evaluated.

### Statistical analysis

Statistical analysis was performed using GraphPad Prism 5.0 (GraphPad Software, Inc., San Diego, CA, USA). The figures were also constructed using GraphPad Prism 5.0. Data are expressed by bar charts or box plots. The Student's t-test or the Mann–Whitney *U* test was applied for statistical analysis, as appropriate. A survival curve was obtained using the Kaplan-Meier method and compared by the log-rank test for each prognostic variable. A multivariate analysis was performed using the Statistical Package for the Social Sciences, version 19 (SPSS Inc., Chicago, IL, USA). A *P*-value of less than 0.05 was considered to be statistically significant.

## SUPPLEMENTARY MATERIALS FIGURE



## Abbreviations

UCB: urothelial carcinoma of the bladder
NMIBC: non-muscle invasive bladder cancer
MIBC: muscle invasive bladder cancer
TURBT: transurethral resection of bladder tumor
KLγ: gamma- Klotho
KLa: alpha-Klotho
KLb: beta-Klotho
FGF: fibroblast growth factor
EMT: epithelial-to-mesenchymal transition
ATCC: American Type Culture Collection
FBS: fetal bovine serum
PBS: phosphate-buffered saline
RT-PCR: reverse transcription-polymerase chain reaction
GAPDH: Glyceraldehyde 3-phosphate dehydrogenase
siRNA: small interfering RNA
IHC: immunohistochemistry
TUNEL: terminal deoxynucleotidyl transferase-mediated dUTP nick end labeling
IQR: interquartile range
LVI: lymphovascular invasion
INF: infiltration
HR: hazard ratio
CI: confidence interval
FGFR: fibroblast growth factor receptor
HCC: hepatocellular carcinoma
