# Supplementary material for: Gamma-Klotho exhibits multiple roles in tumor growth of human bladder cancer
Source: Oncotarget. 2018 Apr 13;9(28):19508–24. doi: 10.18632/oncotarget.24628 (PMC5929405; doi:10.18632/oncotarget.24628)
Supplement: Supplementary file 1 [file oncotarget-09-19508-s001.pdf]

Gamma-Klotho exhibits multiple roles in tumor growth of human bladder cancer

SUPPLEMENTARY MATERIALS

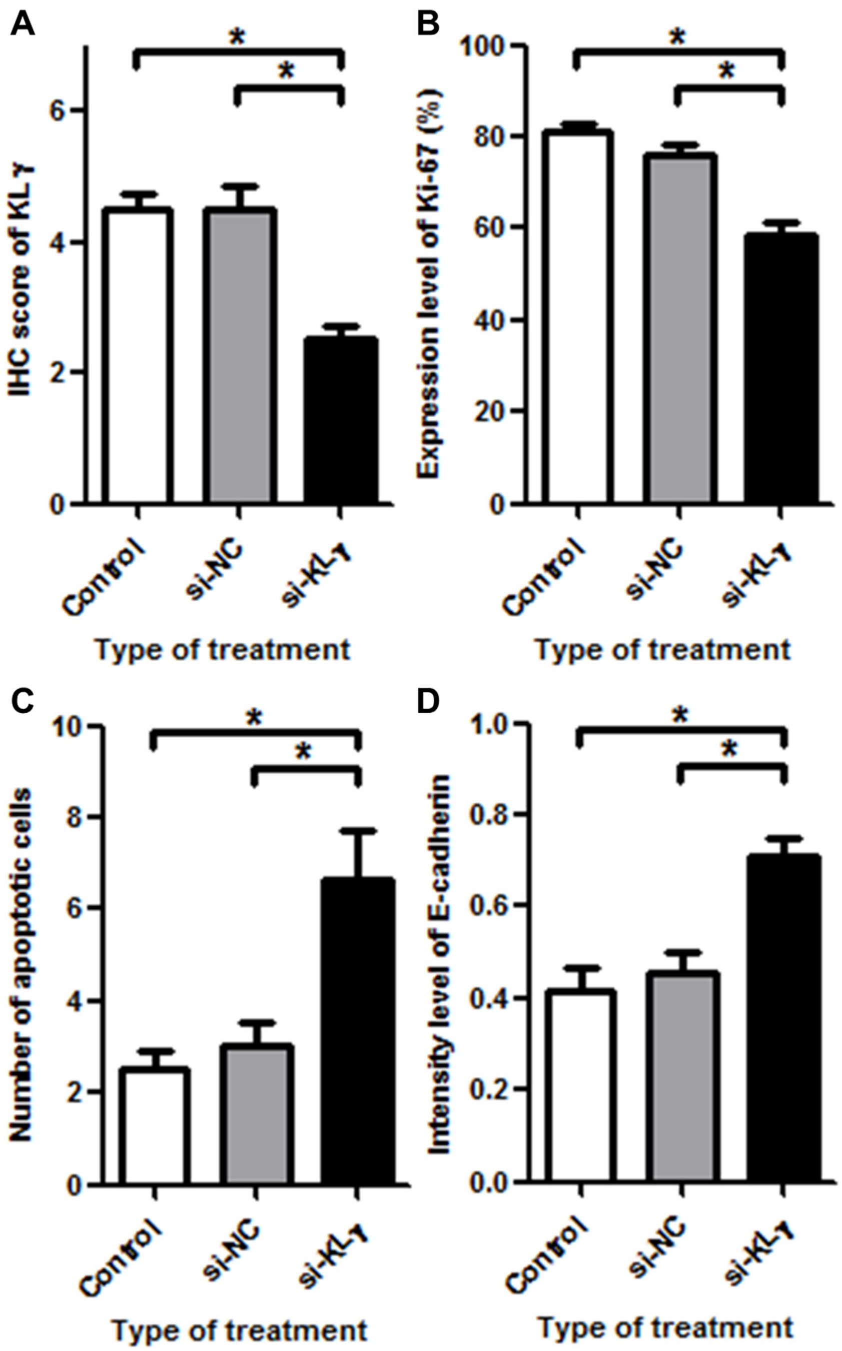

Supplementary Figure 1: Relative expression level of each marker (KLγ, Ki-67, TUNEL, and E-cadherin) in each treatment group. The expression levels of KLγ and Ki-67 decreased in tumors of mice treated with KLγ siRNA. On the other hand, the expression levels of TUNEL and E-cadherin increased in tumors of mice treated with KLγ siRNA (Mann-Whitney U test; * = $P < 0.05$).
